# *Ceratomyxa matosi* n. sp*.* (Myxozoa: Ceratomyxidae) parasitizing the gallbladder of *Boulengerella cuvieri* (Characiformes: Ctenoluciidae) State of Amapá, Brazilian Amazon

**DOI:** 10.1590/S1984-29612024058

**Published:** 2024-10-07

**Authors:** Clemildo Silva Martel, Fábio de Abreu e Souza, Samuel Carvalho Vidal, Abthyllane Amaral de Carvalho, Igor Guerreiro Hamoy, Lilia Suzane de Oliveira Nascimento, Marcela Nunes Videira

**Affiliations:** 1 Programa de Pós-graduação em Ciências Ambientais, Universidade Federal do Amapá – UFAP, Macapá, AP, Brasil; 2 Programa de Pós-graduação em Saúde e Produção Animal na Amazônia, Universidade Federal Rural da Amazônia – UFRA, Belém, PA, Brasil; 3 Programa de Pós-graduação em Biologia de Agentes Infecciosos e Parasitários, Instituto de Ciências Biológicas, Universidade Federal do Pará – UFPA, Belém, PA, Brasil; 4 Laboratório de Genética Aplicada, Universidade Federal Rural da Amazônia – UFRA, Belém, PA Brasil; 5 Laboratório de Morfofisiologia e Sanidade Animal, Universidade do Estado do Amapá – UEAP, Macapá, AP, Brasil

**Keywords:** Parasitism, Brazilian Amazon, fish, Myxozoa, Parasitismo, Amazônia brasileira, peixes, Myxozoa

## Abstract

Myxozoa is a class of the Phylum Cnidaria made up of endoparasites from aquatic habitats. The genus *Ceratomyxa* preferentially infects marine fish, with the gallbladder being the main site parasitized. This study aimed to describe a new species of *Ceratomyxa* found in this organ in *Boulengerella cuvieri* using morphological, morphometric characterization and phylogenetic analysis of 18S rDNA gene sequences. Specimens of *B. cuvieri* were collected, anesthetized, desensitized and biometric measurements were performed. The organs were analyzed under a stereomicroscope and fragments of internal organs were extracted for light microscopy analysis, preserved in 80% ethanol for 18S rDNA gene analysis and fixed in Davidson solution for histological processing. Free spores of *Ceratomyxa* were observed in the gallbladder, in plasmodia with wave-like movements, with the following dimensions: spore width (24.5 ± 0.4) µm, spore length (5.2 ± 0.3) µm, polar capsule width (1.8 ± 0.2) µm, polar capsule length (2.1 ± 0.3) µm, number of polar tubule turns (4-5) and 100% prevalence. Phylogenetic analysis confirmed that *Ceratomyxa matosi* n. sp. is a new species, grouped with other freshwater *Ceratomyxa* species from the Amazon, representing the second description of species of this genus in the state of Amapá.

## Resumo

Myxozoa é uma classe do Filo Cnidaria, constituída por endoparasitos de habitats aquáticos. O gênero *Ceratomyxa* infecta, preferencialmente, peixes marinhos, sendo a vesícula biliar o principal sítio parasitado. Este estudo objetivou descrever uma nova espécie de *Ceratomyxa* encontrada nesse órgão em *Boulengerella cuvieri* usando caracterização morfológica, morfométrica e análise filogenética de sequências do gene 18S rDNA. Exemplares de *B. cuvieri* foram coletados, anestesiados, dessensibilizados e realizada aferição biométrica. Os órgãos foram analisados em estereomicroscópio, e fragmentos de órgãos internos foram extraídos para análise por microscopia de luz, preservado em etanol a 80%, para análise do gene 18S rDNA, e fixados em Davidson para processamento histológico. Por meio de ML, foram observados esporos livres de *Ceratomyxa* na vesícula biliar, em plasmódios com movimentos ondulatórios, com as seguintes dimensões: largura dos esporos (24,5±0,4) µm; comprimento dos esporos (5,2 ± 0,3) µm; largura da cápsula polar (1,8±0,2) µm; comprimento da cápsula polar (2,1 ± 0,3) µm; número de espiras do túbulo polar (4-5) e prevalência de 100%. A análise filogenética confirmou que *Ceratomyxa matosi* n. sp. é uma nova espécie, agrupando-se com outras espécies de *Ceratomyxa* de água doce da Amazônia, representando a segunda descrição de uma espécie desse gênero no estado do Amapá.

## Introduction

The river basins of South America have abundant ichthyofauna, and the richness and diversity of species highlight the importance of these ecosystems, especially the Amazon basin that exhibits one of the greatest freshwater fish diversity worldwide (Reis et al., 2003).

*Boulengerella* species are the most widely distributed in these basins, especially in the Orinoco, Amazon, Tocantins, Pará, and Amapá basins (Vari, 1995). Among the five species identified in this group to date, *Boulengerella cuvieri* Spix & Agassiz, 1829 is important piscivorous species commonly found in the municipality of Ferreira Gomes (State of Amapá), and *B. cuvieri* is the largest species of this genus (Vari, 1995).

Myxozoa Grassé, 1970 is a class of Phylum Cnidaria, which is composed of diverse endoparasites in parasitic relationships with different marine and freshwater species (Kyger et al., 2021). It is divided into two subclasses: Malacosporea and Myxosporea (Fiala et al., 2015). *Ceratomyxa* Thélohan, 1892 is a genus comprising approximately 270 species of myxosporeans that preferentially infect marine fish. The diversity of endoparasites infecting freshwater fish is relatively low, with the gallbladder being the main site of infection and urinary bladder being a rarely infected organ (Eiras et al., 2018).

The diversity of microparasites for the known species, *B. cuvieri*, is very low. *Henneguya pindaibensis* is a microparasite for *B. cuvieri* parasitizing its gills; it was identified using morphological and molecular analyzes after collection from the Pindaíba River, Municipality of Cocalinho, Mato Grosso, Brazil (Úngari et al., 2021).

This work reports a new species of *Ceratomyxa* found in the gallbladder of *B. cuvieri* based on morphological characterization and phylogenetic analysis of *18S rDNA* gene sequences.

## Material and Methods

### Host collection

**Figure 1:**
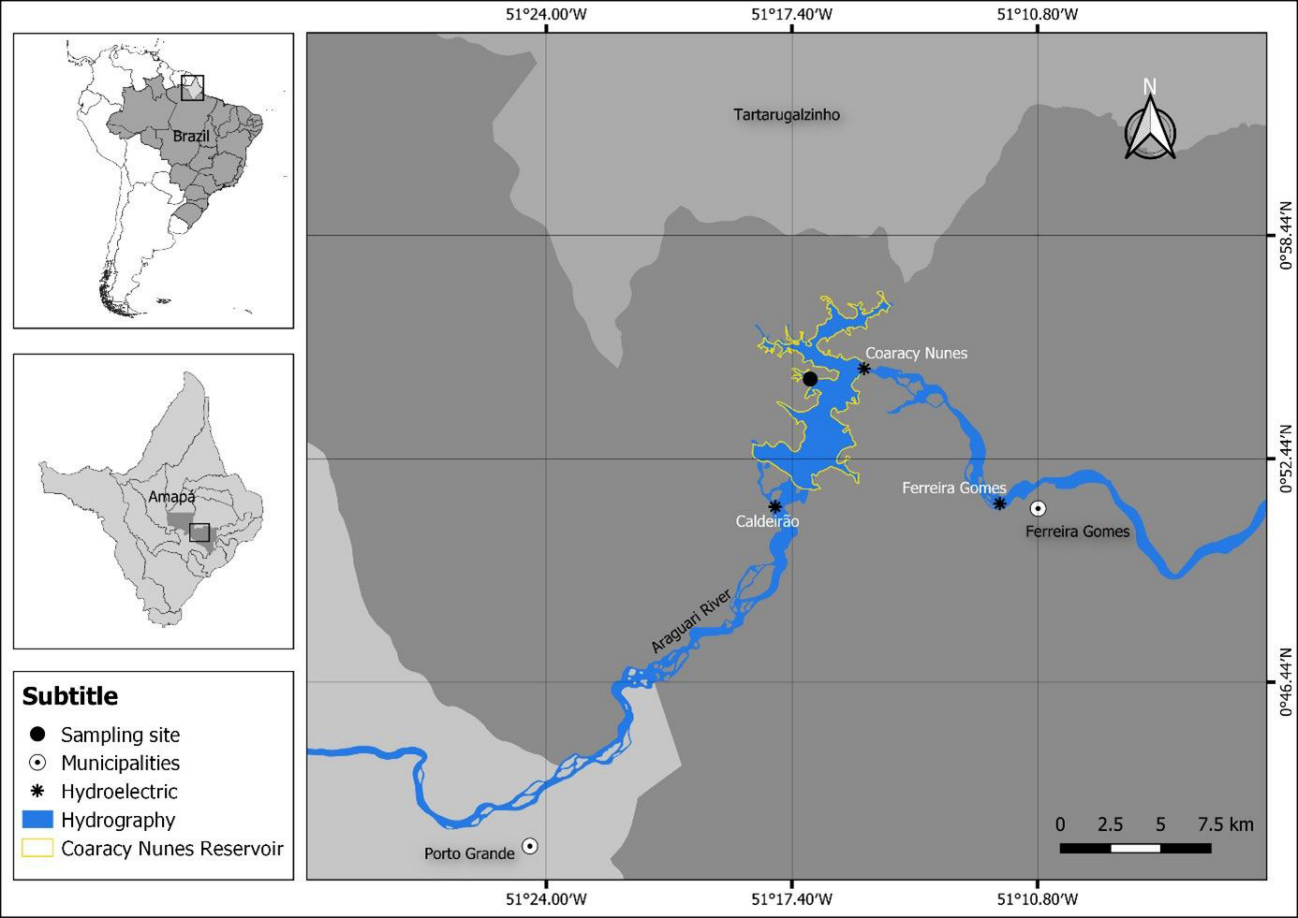
Map with georeferenced collection points in the Reservoir of the Coaracy Nunes Hydroelectric Power Plant (UHCN) located in the municipality of Ferreira Gomes, state of Amapá.

*Boulengerella cuvieri* (n=20) was collected quarterly from the reservoir of the Coaracy Nunes Hydroelectric Power Plant (Figure 1). District of Paredão, Municipality of Ferreira Gomes, State of Amapá (Brazil) from December 2022 to December 2023.

Specimen collection and analysis were coordinated by the Amazon Aquatic Organism Health Research Group at the State University of Amapá (UEAP), approved by the Animal Use Ethics Committee of the Federal Rural University of the Amazon (No. 8323110522), and registered in the Biodiversity Authorization and Information System (SISBIO/ICMBIO, license 50376-1).

The collected fish were transported in suitable containers to the Laboratory of Morphophysiology and Animal Health at UEAP, where they were acclimatized and maintained in specific aquariums for subsequent parasitological analysis.

### Morphological analysis and parasite collection

In the laboratory, the fish were anesthetized (MS-222 Sigma at a concentration of 50 mg/L) and desensitized via spinal sectioning, and biometric measurements were performed. The organs were analyzed under a stereomicroscope, and fragments were extracted for light microscopy (LM), preserved in 80% ethanol for *18S rDNA* gene analysis, and fixed in Davidson solution for standard histological processing. Microparasites were collected and fixed as described by Eiras et al. (2006), and the parasite prevalence was determined as described by Bush et al. (1997).

In the parasitological study of fish, histological processing techniques are fundamental for the analysis of specific tissues. These techniques involve the collection of tissue fragments, fixation in Davidson solution, dehydration in increasing ethanol solutions (70%, 80%, 90%, absolute I, absolute II, and absolute III), diaphanization, and paraffin impregnation to create tissue blocks. These blocks are then cut to obtain 5µm thick histological sections using the Leica RM2125 RTS microtome.

### Molecular and phylogenetic analyses

The collected materials containing microparasites and tissue fragments parasitized with microparasite spores were fixed in 80% ethanol at 4 °C. At the UEAP Molecular Biology of Parasites Laboratory, total DNA from each sample was extracted using the ReliaPrep gDNA Tissue Miniprep System kit (Promega), following the manufacturer's pp>The *18S rDNA* gene fragment was amplified using nested polymserase chain reaction (PCR) on the MyGene MG96G thermocycler (LongGene). The first amplification step was performed using the primers 18E (CTGGTTGATCCTGCCAGT) and 18R (CTACGGAAACCTTGTTACG) (Whipps et al., 2003). The second amplification step was performed using the primers 18E–MC3 (GATTAGCCTGACAGATCACTCCACGA) and 18R-MC5 (CCTGAGAAACGGCTACCACATCCA) (Molnár et al., 2002; Whipps et al., 2003). The PCR products were subjected to electrophoresis on a 1.5% agarose gel in TBE buffer, stained with Safer Dye (Kasvi), and visualized using the Bluegel Electrophoresis System. Successfully amplified samples were purified and sequenced.

A dataset containing 15 sequences of myxosporean species was assembled and compared with the data on GenBank using the Basic Local Alignment Search Tool on the National Center for Biotechnology Information. This dataset composed of the rDNA sequences of both freshwater and marine species was used for phylogenetic analyses. The nucleotide sequences were aligned using ClustalW (Thompson et al., 1997) with the BioEdit program (Hall, 1999), and the unsigned variable regions in the *18S rDNA* datasets were edited.

To determine the phylogenetic relationships among the taxa, maximum parsimony and Bayesian analyses were performed using PAUP 4.0 b10 (Swofford & Sullivan, 2003) and MrBayes 3.1.2 (Ronquist & Huelsenbeck, 2003), respectively. Maximum parsimony analysis was performed using a heuristic search algorithm, which assigned equal weight to transitions and transversions, and insertions and deletions (indels) were considered as missing data. Subsequently, 1,000 bootstrap replications were evaluated, and the confidence level of the most parsimonious tree nodes was calculated (Felsenstein, 2004). Two parallel runs of four simultaneous searches were conducted for Bayesian analysis using the Markov chain Monte Carlo methodology for 5,000,000 generations each, considering one tree every 1,000 generations and disregarding the results of the first 1,250 trees (representing 25% of the samples). The remaining trees (3,750) were used to estimate the confidence level of each node during phylogenetic reconstruction.

For all analyses, DNA sequences of the organisms were directly obtained from GenBank using JMODELTEST 2.0.2, as previously described (Darriba et al., 2012).

## Results

### Morphological description of the spores

**Figure 2:**
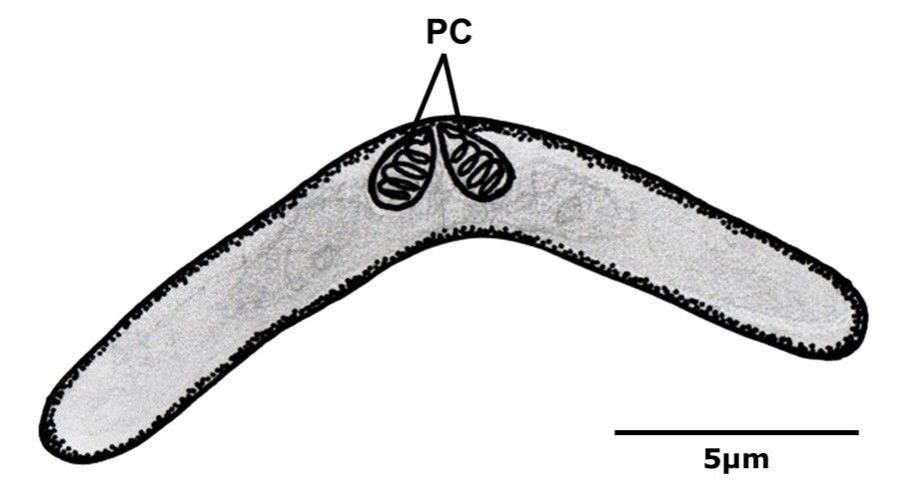
Schematic drawing of the spore frontal view of *Ceratomyxa matosi* n. sp. PC = polar capsule.

*Ceratomyxa matosi* n. sp. (Figure 2) parasitizes the gallbladder of *B. cuvieri*. Using light microscopy, vermiform plasmodia with wave-like movements were observed in the gallbladder fragments containing free spores with elongated and slightly arched structures perpendicular to the suture line and two identical polar capsules, which are common morphological features of genus *Ceratomyxa*.

**Figure 3:**
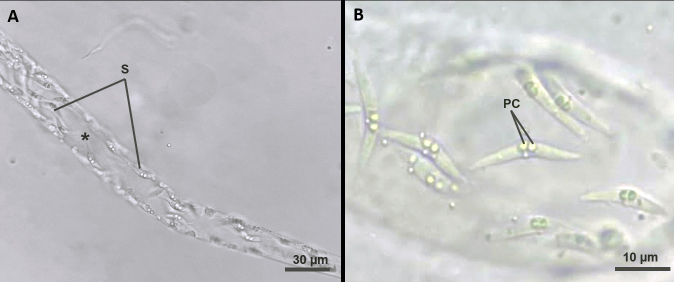
(A) Plasmodia containing *Ceratomyxa matosi* n. sp. showed wave movements when observed with light microscopy. (B) Spores of *Ceratomyxa matosi* n. sp. parasitizing the gallbladder of *Boulengerella cuvieri* Spix & Agassiz, 1829, observed in analyzed samples, alone or in groups. PC = polar capsule; S = spores; Asterisk = plasmodia.

All specimens of *B. cuvieri* collected and analyzed in this study contained spores of *Ceratomyxa matosi* n. sp. in the gallbladder (Figure 3). These spores were observed in all analyzed samples, alone or in groups, with medium dimensions (Table 1) as follows: spore width (24.5 ± 0.4) µm, spore length (5.2 ± 0.3) µm, polar capsule width (1.8 ± 0.2) µm, polar capsule length (2.1±0.3) µm, number of polar tubule coils (4–5). Slides containing histological sections of the gallbladder were stained with hematoxylin and eosin, and coelozoic microparasites were identified.

**Table 1:** Comparative table of measurements (μm) with standard deviation of *Ceratomyxa matosi* n. sp. and other *Ceratomyxa* spp. described in Amazon.

**Species**	**ST**	**SW**	**PCL**	**PCW**	**PA**	**NPT**	**Host**	**Locality**
***Ceratomyxa matosi* n. sp.** **This study**	**24.5 ± 0.4**	**5.2 ± 0.3**	**2.1 ± 0.3**	**1.8 ± 0.2**	**-**	**4-5**	***Boulengerella cuvieri***	**Araguari River, AP**
***C ranunculiformis***	37.6 (32.4–43.9)	4.9 (4–6.6)	2 (1.4–3)	1.9 (1.4–2.4)	165◦ (154–173)	2–3	*Plagioscion squamosissimus*	Grande do Curuai Lake, PA
***C. barbata***	21.7 ± 3.5 (29.9–17.6)	2.9 ± 0.5 (3.8–2.1)	1.6 ± 0.3 (1.1–2.3)	1.4 ± 0.16 (1.5–1.1)	164º ± 10.8º (139º–178º)	3	*Rhaphiodon vulpinus*	Tapajós River, PA
***C. mandii***	31,2 ± 2,3 (26.2–36.3)	4.6 ± 0,5 (3.4–5.5)	1.8 ± 0,3 (1.0–2.5)	1.9 ± 0,3 (1.2–2.4)	162º ± 10,4º (143◦–178º)	3–4	*Pimelodina flavipinnis*	Solimões River, PA
***C.* cf. *fonsecai***	28 ± 1.7 (24.7–31.7)	3.3 ± 0.2 (2.9–3.9)	1.6 ± 0.3 (1.1-2.3)	1.5 ± 0.3 (0.9–2.1)	166º ± 7.43º (146º – 179º)	-	*Hemiodus orthonops*	Paraná River, PR
***C. macapaensis***	22.7 ± 0.3	4.2 ± 0.5	1.8 ± 0.3	1.6 ± 0.1	-	3–4	*Mesonauta festivus*	Piririm River, AP
***C. fonsecai***	28.9 (2.7)	2.6 (0.1)	1.9 (0.3)	1.7 (0.2)	164.8º (8.6º)	3–4	*Hemiodus unimaculatus*	Tocantins River, MA
***C. gracillima***	7.0 ± 0.5 (6.0–8.2)	4.4 ± 0.4 (3.3–5.7)	1.9 ± 0.3 (1.5–2.5)	1.9 ± 0.3 (1.5–2.5)	36.6º ± 2.9º (35º– 40º)	2–3	*Brachyplatystoma rousseauxii*	Tapajós River, PA
***C. brasiliensis***	41.2 ± 2.9 (37.1–47.6)	6.3 ± 0.6 (5.1–7.5)	2.6 ± 0.3 (2–3.3)	2.5 ± 0.4 (1.8–3.7)	147º	3–4	*Cichla monoculus*	Tapajós River, PA
***C. vermiformis***	8.4 ± 0.4 (7.9–9.3)	4.5 ± 0.2 (4.2–4.8)	2.7 ± 0.1 (2.5–2.9)	2.7 ± 0.1 (2.5–2.9)	30.2º ± 6.6º (22º– 43º)	3–4	*Colossoma macropomum*	Tapajós River, PA
***C. amazonensis***	15.8 ± 0.4 (15.0–16.7)	7.0 ± 0.3 (6.2–7.6)	3.2 ± 0.3 (2.4–3.6)	2.6 ± 0.2 (2.4–2.9)	105º– 115º	3–4	*Symphysodon discus*	Negro River, AM
***C. microlepis***	35.5 ± 0.9	5.2 ± 0.4	2.2 ± 0.3	2.2 ± 0.3	58º – 60º	5–6	*Hemiodus microlepis*	Trombetas River, PA
*C. mylei (syn. *Meglitschia mylei*)	*-*	-	2.1 ± 0.3	2.1 ± 0.3	-	5–6	*Myleus rubripinnis*	Sapuruá Lake, AM

IS: infection site. ST: spore length, SW: spore width, PCL: polar capsule length, PCW: polar capsule width, PA: posterior angle, NPT: number of polar tubule coils; IS: infection site.

* The original description was made as a species of the genus *Meglitschia* and presents a measurement pattern different from that used for the genus *Ceratomyxa* (Azevedo et al., 2011).

### Taxonomic summary

Kingdom: Animalia Linnaeus, 1758

Phylum: Cnidaria Hatscheck, 1888

Class: Myxozoa Grassé, 1970

Subclass: Myxosporea Bütschli, 1881

Order: Bivalvulida Shulman, 1959

Family: Ceratomyxidae Doflein, 1899

Genus: *Ceratomyxa* Thélohan, 1892

Species: *Ceratomyxa matosi* n. sp.

Infection site: Coelozoic plasmodia with spores of *Ceratomyxa matosi* n. sp. distributed in the host gallbladder.

Locality: Reservoir of the Coaracy Nunes hydroelectric power plant in the District of Paredão, Municipality of Ferreira Gomes, State of Amapá, Brazil (N00°54’34.3’’, W051°16’54.2’’)

Prevalence: 100% (n=20).

Species deposition: A glass slide with hematoxylin and eosin-stained spores has been deposited into the Zoological Collection of the Amazon Research Institute (INPA – CND 000103) in Manaus, Amazonas, Brazil.

DNA sequence: The *18S rDNA* gene sequence (549 bp) has been deposited in GenBank under the accession no. PP791852.

Etymology: The specific epithet for this species was given in honor to Dr. Edilson Rodrigues Matos (*in memoriam*), an exceptional myxozoan researcher in the Brazilian Amazon.

### Phylogenetic and molecular analyses

The partial sequence of *C. matosi* n. sp. containing 549 bases pairs of SSU rDNA gene determined in this study has been deposited in GenBank under the accession number PP791852. *Ceratomyxa matosi* n. sp. showed the greatest genetic proximity (Table 2) to *Ceratomyxa vermiformis* Adriano & Okamura, 2017 (6%), followed by *Ceratomyxa mandii* Araújo, Adriano, Franzolin, Zatti & Naldoni, 2022 (7%) and *Ceratomyxa gracillima* Zatti, Atkinson, Maia, Bartholomew & Adriano, 2017 (8%) (Adriano & Okamura, 2017; Zatti et al., 2017a; Araújo et al., 2022)*.*

**Table 2:** The uncorrected p-distances recorded between pairs of *Ceratomyxa* spp. that comprise the clade of registered *Ceratomyxa* spp. around the world.

**Species**	**1**	**2**	**3**	**4**	**5**	**6**	**7**	**8**
**1. *Ceratomyxa_matosi* n.sp.**	-	-	-	-	-	-	-	-
2. *Ceratomyxa vermiformis* KX278420	0.067	-	-	-	-	-	-	-
3. *Ceratomyxa gracílima* KY934184	0.081	0.032	-	-	-	-	-	-
4. *Ceratomyxa mandii* MZ504285	0.070	0.043	0.021	-	-	-	-	-
5. *Ceratomyxa pallida* KR086361	0.191	0.175	0.175	0.171	-	-	-	-
6. *Ceratomyxa tunisiensis* KT013098	0.165	0.150	0.148	0.151	0.041	-	-	-
7. *Ceratomyxa leatherjacketi* KM273028	0.198	0.156	0.152	0.157	0.088	0.075	-	-
8. *Ceratomyxa shasta* AF001579	0.283	0.223	0.229	0.237	0.238	0.240	0.253	-
9. *Ceratomyxa gasterostea* KF751186	0.280	0.228	0.231	0.251	0.251	0.240	0.252	0.155

**Figure 4:**
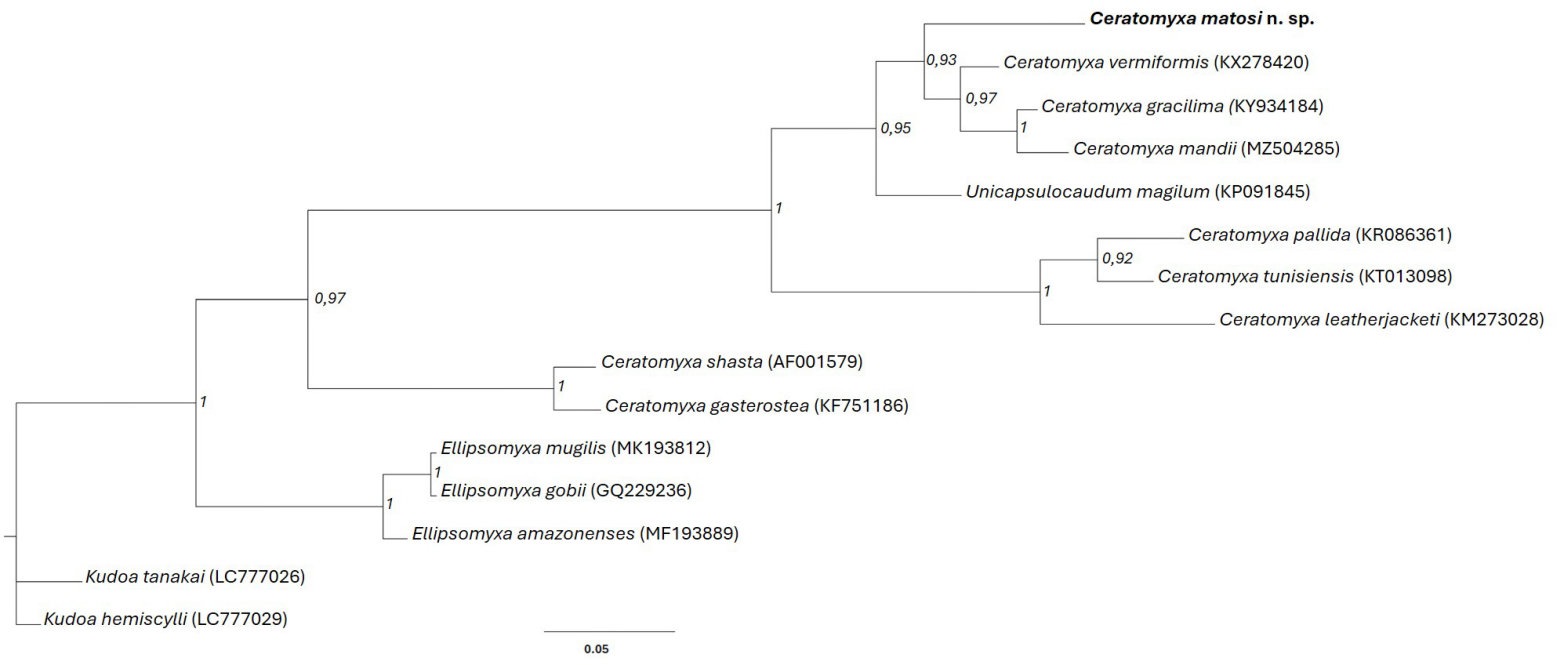
Phylogenetic tree generated by Bayesian inference (BI) through partial alignment of *Ceratomyxa matosi* n. sp. with SSU r DNA gene sequences of select myxozoan species. Node numbers are indicated for posterior probabilities values calculated by (BI).

Phylogenetic analysis revealed that *C. matosi* n. sp. exhibited monophyletic behavior, where this new species was grouped with other *Ceratomyxa* spp. in the Amazon (Figure 4). In subclade A of the *C. matosi* n. sp. group, *C. vermifomis*, *C. gracillima*, and *C. mandii* were observed with strong nodal support (Adriano & Okamura, 2017; Zatti et al., 2017a; Araújo et al., 2022).

## Discussion

*Ceratomyxa matosi* n. sp. is the second most common species of genus *Ceratomyxa* in the state of Amapá, following *Ceratomyxa macapaensis* Bittencourt, Silva, Hamoy, Carvalho, Silva, Videira & Matos, 2022 the first described species this genus in the State of Amapá, and both species parasitize the gallbladder of their host (Bittencourt et al., 2022).

Plasmodia-containing *Ceratomyxa matosi* n. sp. exhibited wave-like movements in fresh ML. Adriano & Okamura (2017) suggested that such movements are due to the presence of mitochondria surrounding the plasmodia, a cytoskeleton with abundant actin, and formation of microtubules. This movement is the same as that described for the plasmodia of *Ceratomyxa fonsecai* Silva, Carvalho, Hamoy & Matos, 2020 and *C. macapensis* (Silva et al., 2020; Bittencourt et al., 2022).

Morphological analyzes of the spores of *Ceratomyxa matosi* n. sp. compared to other freshwater *Ceratomyxa* spp. in the Amazon, revealed greater affinity with *C. vermiformis* (Adriano & Okamura, 2017) parasitizing *Colossoma macropomum* (tambaqui), *C. gracillima* (Zatti et al., 2017a, b) parasitizing the Amazon catfish *Brachyplatystoma rousseauxii*, and *C. mandii* (Araújo et al., 2022) parasitizing *Pimelodina flavipinnis* (mandi). The host gallbladder was the infection site for all these parasites.

Comparative dimensional data of *Ceratomyxa matosi* n sp. revealed that the spores of this species exhibited greater thickness (24.5±0.4 µm) than the spores of *C. vermiformis* (8.4 ± 0.4 µm) and *C. gracilima* (7.0 ± 0.5 µm) but lesser thickness than the spores of *C. mandii* (31.2 ± 2.3 µm). The length of *Ceratomyxa matosi* n. sp. (5.2 ± 0.3 µm) was longer than those of the three parasite species, *C. vermiformis* (4.5 ± 0.2 µm), *C. gracilima* (4.4 ± 0.4 µm), and *C. mandii* (4.6 ± 0,5 µm).

Lengths and widths of the polar capsules of *Ceratomyxa matosi* n. sp. (2.1 ± 0.3/1.8 ± 0.2 µm), *C. gracílima* (1.9 ± 0.3/1.9 ± 0.3 µm), and *C. mandii* (1.8 ± 0.3/1.9 ± 0.3 µm) were not very different from each other, but the difference was quite significant between the identified taxon in this study and *C. vermiformis* (2.7 ± 0.1/2.7 ± 0.1 µm).

*Ceratomyxa matosi* n. sp. sporoplasm is binucleate, with each polar capsule containing a filament with 4–5 coils oblique to the longitudinal axis that is longer than those of other similar species: *C. vermiformis* (3–4 coils), *C. gracílima* (2–3 coils), and *C. mandii* (3–4 coils). In *C. mandii*, the sutural line is straight, with smooth and slightly thin valves at both ends and subspherical polar capsules (Araújo et al., 2022). *C. gracillima* has spherical polar capsules equal in size and located anteriorly and adjacent to the straight suture (Zatti et al., 2017a). In *C. vermiformis*, the two valves are elongated, resembling unequal size appendages that taper approximately halfway along their length (Adriano & Okamura, 2017).

The phylogenetic arrangement of *Ceratomyxa* spp. was the same as that reported by Fiala et al. (2015). *Ceratomyxa* spp. exhibit undefined phylogenetic relationships. Moreover, presence of many species with long arms on trees indicates the rapid evolution of *Ceratomyxa* spp. compared to other species in freshwater environments. Overall, *Ceratomyxa matosi* n. sp. exhibited monophyletic behavior, where it grouped into a subclade with other species in the Brazilian Amazon.

## Conclusions

The morphological and molecular data confirmed *Ceratomyxa matosi* n. sp. as a new species of Class Myxozoa parasitizing the gallbladders of *B. cuvieri*, a widely distributed freshwater fish. However, further studies are necessary to explore the evolutionary relationships among different Myxozoa species in the Amazon.
